# Synthesis and enzymatic evaluation of 2- and 4-aminothiazole-based inhibitors of neuronal nitric oxide synthase

**DOI:** 10.3762/bjoc.5.28

**Published:** 2009-06-04

**Authors:** Graham R Lawton, Haitao Ji, Pavel Martásek, Linda J Roman, Richard B Silverman

**Affiliations:** 1 Department of Chemistry, Center for Molecular Innovation and Drug Discovery, and Chemistry of Life Processes Institute, Northwestern University, Evanston, Illinois 60208-3113 (USA); 2 Department of Biochemistry, University of Texas Health Science Center, San Antonio, Texas (USA); 3 Department of Pediatrics and Center for Applied Genomics, 1st School of Medicine, Charles University, Prague, Czech Republic

**Keywords:** 2-aminothiazole, 4-aminothiazole, nitric oxide synthase inhibitor, nNOS

## Abstract

Highly potent and selective inhibitors of neuronal nitric oxide synthase (nNOS) possessing a 2-aminopyridine group were recently designed and synthesized in our laboratory and were shown to have significant in vivo efficacy. In this work, analogs of our lead compound possessing 2- and 4-aminothiazole rings in place of the aminopyridine were synthesized. The less basic aminothiazole rings will be less protonated at physiological pH than the aminopyridine ring, and so the molecule will carry a lower net charge. This could lead to an increased ability to cross the blood-brain barrier thereby increasing the in vivo potency of these compounds. The 2-aminothiazole-based compound was less potent than the 2-aminopyridine-based analogue. 4-Aminothiazoles were unstable in water, undergoing tautomerization and hydrolysis to give inactive thiazolones.

## Introduction

Neuronal nitric oxide synthase (nNOS) is the constitutive isoform of nitric oxide synthase (NOS) found in the CNS. It is believed to play a role in many neurological diseases, including Parkinson’s disease [1], Alzheimer’s disease [2], damage due to stroke [3], and cerebral palsy caused by pre-natal hypoxia [4]. To fully explore the role nNOS plays in these and other diseases, and to design inhibitors with therapeutic value, nNOS must be inhibited selectively without inhibition of the other isoforms, inducible NOS (iNOS) and endothelial NOS (eNOS); inhibition of eNOS could lead to side effects such as hypertension [5].

Designing therapeutically useful nNOS-selective inhibitors is a difficult task as the substrate for all three isoforms is L-arginine, and so they have similar active sites. In recent years, we have developed several potent and highly selective inhibitors of nNOS that have solved this problem by exploiting subtle differences among the isoforms [6]. In addition, the active site of nNOS is polar with multiple acidic groups, and so most inhibitors that bind with high potency are polar with multiple basic groups. In general, highly charged, hydrophilic molecules do not diffuse passively across the blood-brain barrier (BBB), thus limiting the concentration of inhibitor in the CNS [7].

**Figure 1:**
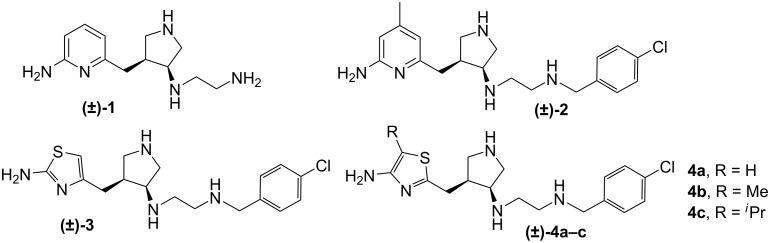
Lead compounds **1** and **2**; 2- and 4-aminothiazole analogs **3** and **4a**-**c**.

Recently, a new strategy for fragment-based de novo design, called fragment hopping, was described and utilized to design **1** (Figure 1), a new nNOS-selective inhibitor [8], which showed nanomolar nNOS inhibitory potency and more than 1000-fold selectivity over eNOS. Lead compound **1** was then evolved into highly potent and selective inhibitor **2** with better drug-like properties [9]. Intravenous administration of **2** resulted in significant protection against neuronal damage in a cerebral palsy rabbit model [10].

**Figure 2:**
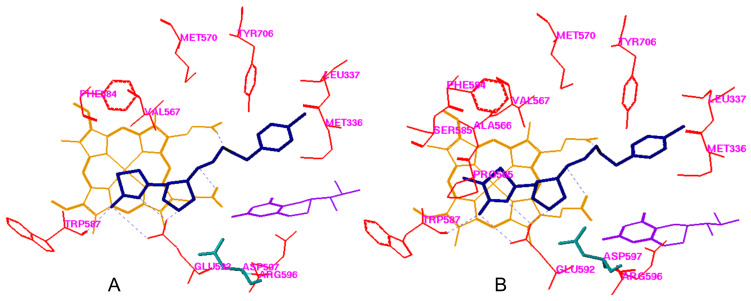
A: The docking conformation of **3** in the active site of rat nNOS; B: The docking conformation of **4b** in the active site of rat nNOS. Cofactors heme and H_4_B are shown in orange and purple, respectively.

Replacement of basic functional groups of our lead molecule with less basic groups will reduce the overall charge on the molecule and should increase BBB penetration, leading to greater in vivo efficacy. The conjugate acid of the ring nitrogen of the aminopyridine has a pK_a_ ≈ 7 [11], therefore, the ring will be partially protonated and only partially charged in biological systems. Crystal structures of **2** bound to the active site of nNOS show that the aminopyridine group interacts with a glutamate residue (Glu592, rat nNOS), presumably via hydrogen bonding and electrostatic interactions [8,12]. The aminopyridine ring nitrogen must be protonated for this interaction to occur. Replacement of the aminopyridine with less basic aminothiazoles (**3** and **4**), whose ring nitrogens have pK_a_ values ≈ 6, should reduce the overall charge on the molecule at physiological pH for more efficient bioavailability. In the acidic environment of the nNOS active site, however, protonation should occur to allow the aminothiazole to interact with the active site glutamate and maintain tight binding. The methyl group at the 4 position on the aminopyridine ring contributes to binding through an interaction with a hydrophobic pocket in the nNOS active site. The R-groups at the 5-position of **4** should allow us to probe the hydrophobic binding pocket defined by P565, A566, V567, and F584 in the substrate binding site and optimize this interaction. Figure 2 shows the docking mode for **3** and **4a** in rat nNOS.

**Scheme 1:**
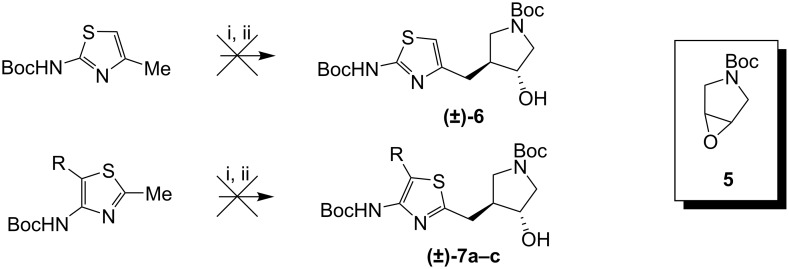
Attempts to open epoxide **5** with deprotonated aminothiazoles. i) *n*-BuLi, 2 equiv, THF, −78 °C; ii) **5**, THF, −78 °C to rt.

Initially, attempts were made to construct **3** and **4** using chemistry analogous to that used to construct **2** (Scheme 1). Briefly, Boc protected 2-amino-4-methylthiazoles, and 4-amino-2-methylthiazoles were treated with *n*-butyllithium, and then epoxide **5** was added in an attempt to form *trans* alcohols **6** and **7**. However, this method failed to give any of the desired products.

**Scheme 2:**
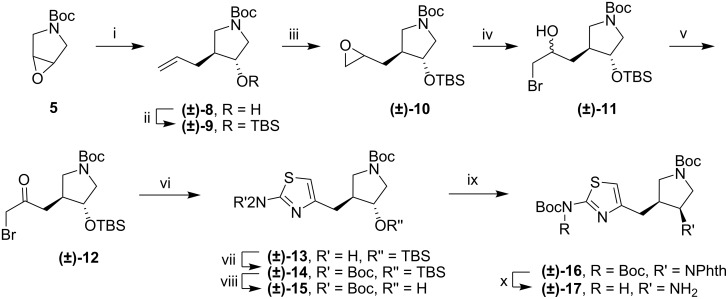
Assembly of 2-aminothiazole fragment. i) AllylMgBr, ether, 0 °C, 15 min.; ii) TBSCl, imidazole, DMF, 35 °C, 16 h; iii) m-CPBA, rt, 48 h; iv) LiBr, AcOH, THF, rt, 16h; v) (COCl)_2_, DMSO, TEA, CH_2_Cl_2_, −78 °C, 1 h; vi) thiourea, EtOH, reflux, 5 h; vii) Boc_2_O (2.5 equiv), DMAP, THF, rt, 16 h; viii) TBAF, THF, rt 16 h; ix) PPh_3_, DIAD, phthalimide, THF, rt, 16 h; x) H_2_NNH_2_ (aq), MeOH, rt, 16 h, then 2N HCl, rt, 30 min.

A successful route to 2-aminothiazoles is outlined in Scheme 2. Epoxide **5** was opened with allylmagnesium bromide, and the resulting alcohol was protected as a TBS ether. The double bond was converted to an epoxide, which was then opened with bromide under acidic conditions [13]. A mixture of diastereomers was formed, but both were oxidized to α-bromoketone **12**. Condensation with thiourea gave the 2-aminothiazole (**13**). Diprotection of the amine with Boc groups and deprotection of the TBS ether gave *trans*-alcohol **15**. A Mitsunobu reaction was used to install a nitrogen atom in the form of a phthalimide group with the desired *cis*-stereochemistry. The amine was deprotected, but that resulted in the loss of one of the Boc groups to give **17**.

**Scheme 3:**
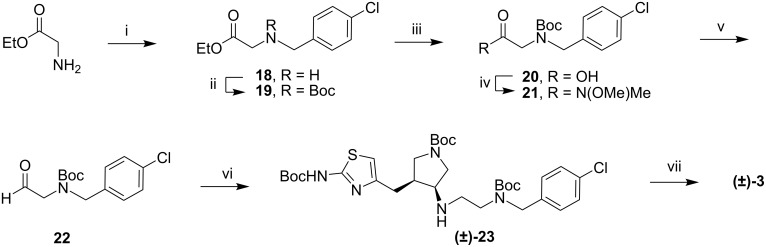
Synthesis of compound **3**. i) 4-chlorobenzylchloride, EtOH, reflux, 4 h; ii) Boc_2_O, TEA, MeOH, 3 h; iii) 1 N NaOH, MeOH, rt, 4 h; iv) EDC, HOBt, TEA, HN(OMe)Me·HCl, CH_2_Cl_2_, 16 h; v) LAH, THF, 0 °C, 1 h; vi) (±)-17, CH_2_Cl_2_, NaHB(OAc)_3_, 1 h; vii) 4N HCl, dioxanes, rt, 16 h.

Ethyl glycinate was alkylated with *p*-chlorobenzyl chloride to give secondary amine **18**, which was protected with a Boc group. The ester was hydrolyzed and converted to the Weinreb amide. Reduction with LAH gave aldehyde **22** (Scheme 3).

Compounds **17** and **22** were condensed to form an imine prior to the addition of NaHB(OAc)_3_, giving secondary amine **23**. Removal of the Boc groups with acid gave **3** as a tetrahydrochloride salt.

**Scheme 4:**
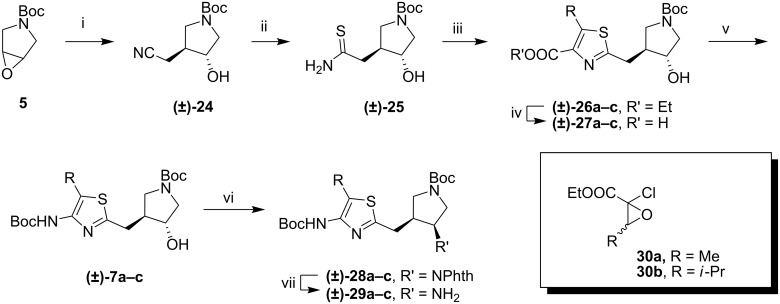
Assembly of the 4-aminothiazole fragments. i) LiCH_2_CN, THF, 0 °C, 4 h; ii) (NH_4_)_2_S (aq), MeOH, 16 h; iii) ethyl brompyruvate (for R = H) or **30**, MeOH, reflux, 5 h, then DIEA, Boc_2_O, rt, 16 h; iv) 1N NaOH (aq), MeOH, rt, 16 h; v) DPPA, TEA, 3 Å mol. sieves, *t*-BuOH, reflux, 16 h; vi) PPh_3_, DIAD, phthalimide, THF, rt, 16 h; vii) H_2_NNH_2_ (aq), MeOH, rt, 16 h, then 2N HCl, rt, 30 min.

The 4-aminothiazoles were constructed as shown in Scheme 4. Treatment of acetonitrile with LDA followed by addition of epoxide **5** gave *trans*-alcohol **24** [14]. The nitrile group was converted to a thioamide using ammonium sulfide [15]. The thioamide was condensed with either ethyl bromopyruvate or an epoxide (**30**) [16]. Condensation produces an equivalent of acid, which was sufficient to cleave the Boc group. Buffering the reaction resulted in incomplete conversion to the thiazole because acidic conditions are necessary to catalyze the final dehydration step in the reaction [17]. However, the problem was solved by simply neutralizing the mixture on completion of the reaction and reprotecting the amine. The resulting esters (**26a**, R = H; **26b**, R= Me; **26c**, R = *i*-Pr) were hydrolyzed, and a Curtius rearrangement performed in *tert*-butanol gave the protected 4-aminothiazoles (**7a**-**c**) [18]. Unlike the case of the aminopyridine analogues [19], the aminothiazole does not need to be diprotected to allow the Mitsunobu reaction with phthalimide as the nucleophile to proceed (**28a**-**c**). This is presumably because the thiazole nitrogen is less nucleophilic. Cleavage of the phthalimide group gave amines **29a**-**c**.

**Scheme 5:**
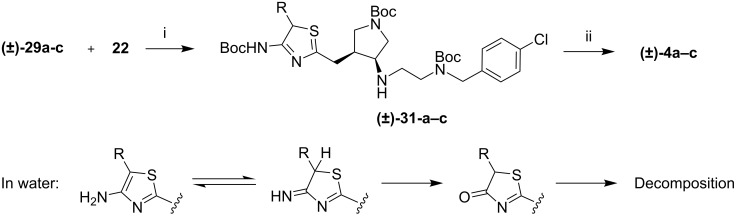
Synthesis of inhibitor **4a**-**c**. The 4-aminothiazoles were not stable in water undergoing tautomerization and hydrolysis. i) MeOH, 30 min, then NaHB(OAc)_3_, rt, 1 h; ii) 4N HCl, dioxanes, rt, 16 h.

The syntheses of **4a**-**c** were completed by reductive amination followed by removal of the Boc groups (Scheme 5). Although the final deprotection did give the desired product, as evidenced by mass spectrometry, on addition of water, the product decomposed. The 4-aminothiazole tautomerized to the thiazoline, which was then hydrolyzed [20]. Test reactions on model 4-aminothiazoles prepared by an analogous route showed that none of the desired product remains in aqueous solution. Further degradation occurred following hydrolysis, and the products could not be identified.

## Results and Discussion

Compound **3** was tested for in vitro activity against rat nNOS [21], bovine eNOS [22], and murine iNOS [23] using a hemoglobin capture assay [24], giving *K*_i_ values of 10 μM, 1 mM and 50 μM, respectively. This corresponds to a significant loss in potency toward nNOS relative to lead compound **2** [*K*_i_(nNOS) = 0.085 μM, *K*_i_(eNOS) = 85 μM, *K*_i_(iNOS] = 9 μM), suggesting that the aminopyridine ring is critical for high affinity binding. It has been shown that the addition of electron-withdrawing groups to a 2-aminopyridine significantly decreases NOS affinity [25]. This is possibly the result of a lowering of the p*K*_a_ such that the ring nitrogen is insufficiently protonated to interact with the glutamate. We had hoped that the high acidity of the active site would protonate the aminothiazole so that it would interact well with the glutamate residue. This may not be the case. An alternative explanation for the loss of potency is that the 2-aminothiazole ring is much smaller than 2-amino-4-methylpyridine. The methyl group at the 4-position of the pyridine ring contributes a significant amount to binding, resulting in a 4-fold increase in potency. There is no functionality in the 2-aminothiazoles to provide a similar hydrophobic interaction. If the 4-aminothiazoles had been stable, the alkyl group in the 5-position could have contributed to binding to restore some of the lost potency.

## Conclusion

2-Aminothiazole-based nNOS inhibitor **3** was synthesized via a condensation reaction between thiourea and the appropriate α-bromoketone. However, the inhibitor was less potent than the aminopyridine lead. Nonetheless, the synthetic methodology is useful for the construction of this ring system. 4-Aminothiazole-based inhibitors with various alkyl groups at the thiazole 5-position could be synthesized, but proved to be unstable in aqueous medium. This is a valuable insight for others contemplating this ring system for biological studies. Unfortunately, the use of an aminothiazole in place of the aminopyridine moiety is not a beneficial modification in the case of our nNOS inhibitors. Alternative methods to reduce the overall charge on the molecule are under investigation.

## Supporting Information

Supporting Information features full experimental details for all synthetic steps, characterization of intermediates (^1^H NMR, ^13^C NMR, ESMS), details of Autodock analysis, details of in vitro enzyme assay, and HPLC chromatograms for determining purity of compound **3**.

File 1 Experimental and analytical data
