# Supplementary material for: Synthesis and enzymatic evaluation of 2- and 4-aminothiazole-based inhibitors of neuronal nitric oxide synthase
Source: Beilstein J Org Chem. 2009 Jun 4;5:28. doi: 10.3762/bjoc.5.28 (PMC2707017; doi:10.3762/bjoc.5.28)
Supplement: File 1 — Experimental and analytical data [file Beilstein_J_Org_Chem-05-28-s001.doc]

**Supporting Information**

**for**

Synthesis and enzymatic evaluation of 2- and 4-aminothiazole-based inhibitors of neuronal nitric oxide synthase

Graham R. Lawton,^1^ Haitao Ji,^1^ Pavel Martásek,^2,3^ Linda J. Roman,^2^ and Richard B. Silverman*^,1^

Address: ^1^Department of Chemistry, Center for Molecular Innovation and Drug Discovery, and Chemistry of Life Processes Institute, Northwestern University, Evanston, Illinois 60208-3113 (USA), ^2^Department of Biochemistry, University of Texas Health Science Center, San Antonio, Texas (USA) and ^3^Department of Pediatrics and Center for Applied Genomics, 1^st^ School of Medicine, Charles University, Prague, Czech Republic

Email: Richard B. Silverman - r-silverman@northwestern.edu

*Corresponding author

# Experimental and analytical data

Table of Contents

S2. Experimental Section.

S22. Autodock Analysis

S23. In vitro enzyme assay

S24. HPLC analysis of Compound **3**, Chromatographic Conditions 1

S25. HPLC analysis of Compound **3**, Chromatographic Conditions 2

**Experimental Section**

**General Methods.** Proton nuclear magnetic resonance spectra (^1^H NMR) were recorded in deuterated solvents on a Varian Inova 500 (500 MHz) spectrometer. Chemical shifts are reported in parts per million (ppm, δ) relative to tetramethylsilane (δ 0.00). ^1^H NMR splitting patterns are designated as singlet (s), doublet (d), triplet (t), quartet (q). Splitting patterns that could not be interpreted or easily visualized were recorded as multiplet (m) or broad (br). Coupling constants are reported in Hertz (Hz). Proton-decoupled carbon (^13^C NMR) spectra were recorded on a Varian Inova 500 (125 MHz) spectrometer and are reported in ppm using the solvent as an internal standard (CDCl_3_, δ 77.23). NMR spectra recorded in D_2_O were not normalized. In many cases, the presence of rotamers made the NMR spectra complex. In the case of two peaks that are clearly a pair of rotamers, but are too far apart for an average to accurately represent the spectrum, the pair is written enclosed in parentheses, or the presence of rotamers is indicated. Electrospray mass spectra (ESMS) were obtained using an LCQ-Advantage with methanol as the solvent in positive ion mode, unless otherwise stated. For most compounds, ^1^H and ^13^C NMR and ESMS data are presented.

All chemical reagents were purchased from Aldrich and were used without further purification unless stated otherwise. NADPH, calmodulin, and human ferrous hemoglobin were also obtained from Sigma-Aldrich. Tetrahydrobiopterin (H_4_B) was purchased from Alexis Biochemicals. HEPES, DTT, and some conventional organic solvents were purchased from Fisher Scientific.

Tetrahydrofuran (THF) was distilled from sodium and benzophenone as the indicator prior to use. Methylene chloride (CH_2_Cl_2_) was distilled from calcium hydride prior to use, if dry solvent was required. Dimethylformamide (DMF) was purchased as an anhydrous solvent and used directly.

***tert*-Butyl 6-oxa-3-azabicyclo[3.1.0]hexane-3-carboxylate (5)** To a solution of 3-pyrroline (765 μL, 10 mmol, 65% pure, Fluka) in methanol (30 mL) at 0 °C was added di-*tert*-butyldicarbonate (Boc_2_O) (2.4 g, 11 mmol). The mixture was stirred for 20 h. The solvent was removed *in vacuo*, and the residue was dissolved in CH_2_Cl_2_ (30 mL) and cooled to 0 °C. mCPBA (1.9 g, 11 mmol, 70% pure) was added, and the mixture was stirred for 44 h. 20% NaS_2_O_3_ (20 mL) was added, and the mixture was stirred vigorously for 30 min. The mixture was separated, and the organic layer was washed with saturated NaHCO_3_ (20 mL), 20% NaS_2_O_3_ (20 mL), saturated NaHCO_3_ (20 mL), and brine (20 mL), dried over Na_2_SO_4_ and concentrated *in vacuo*. The crude product was purified using flash column chromatography (silica gel, ethyl acetate/hexanes, 2:3) to afford **5** as a colorless oil (870 mg, 4.7 mmol, 72%, based on maximum possible yield). ^1^H NMR (500 MHz, CDCl_3_) δ 3.86–3.67 (m, 2H), 3.32 (dd, *J* = 6, 13 Hz, 4H), 1.45 (s, 9H); ^13^C NMR (125 MHz, CDCl_3_) δ 155.0, 80.0, (55.9 + 55.4), (47.6 + 47.2), 28.7.

***tert*-Butyl 3-allyl-4-hydroxypyrrolidine-1-carboxylate (8)** A flame-dried 3-necked flask equipped with stir bar and addition funnel was charged with dry ether (20 mL) and allyl magnesium bromide (11 mL, 1 M solution in ether, 11 mmol). The mixture was cooled to 0 °C. A solution of **5** (920 mg, 5 mmol) in dry ether (20 mL) was added dropwise via the addition funnel. A white precipitate was formed immediately on addition. After the addition was complete, the mixture was stirred for a further 15 min at 0 °C then quenched by dropwise addition of saturated NH_4_Cl solution (25 mL). The layers were separated, and the aqueous layer was further extracted with ether (2 × 10 mL). The organic layers were combined, dried over Na_2_SO_4_ and concentrated *in vacuo.* The crude product was purified using flash column chromatography (silica gel, ethyl acetate/hexanes, 1:2) to afford **8** as a colorless oil (1.09 g, 4.8 mmol, 96%). ^1^H NMR (500 MHz, CDCl_3_) δ 5.80 (m, 1H), 5.07 (m, 2H), 4.06 (m, 1H), 3.57 (m, 2H), 3.23 (m, 1H), 3.06 (m, 1H), 2.28–2.04 (m, 3H), 1.46 (s, 9H); ^13^C NMR (125 MHz, CDCl_3_) δ 155.0, 135.8, 116.7, 79.7, (74.5 + 73.8), 52.7, 49.2, (45.6 + 45.0), 35.8, 28.7; ESMS 228 (M + H)^+^.

***tert*-Butyl 3-allyl-4-(*tert*-butyldimethylsilyloxy)pyrrolidine-1-carboxylate (9)**. A solution of **8** (130 mg, 0.57 mmol), TBSCl (107 mg, 0.72 mmol) and imidazole (95 mg, 1.4 mmol) in anhydrous DMF (5 mL) was stirred at 40 °C for 16 h. The solvent was removed *in vacuo*, and the crude product was purified using flash column chromatography (silica gel, ethyl acetate/hexanes, 1:9) to afford **9** as a colorless solid (181 mg, 0.53 mmol, 93%). ^1^H NMR (500 MHz, CDCl_3_) δ 5.71 (m, 1H), 4.99 (m, 2H), 3.89 (m, 1H), 3.58–3.44 (m, 2H), 3.10–2.93 (m, 2H), 2.19 (m, 1H), 2.03 (m, 1H), 1.91 (m, 1H), 1.42 (s, 9H), 0.84 (s, 9H), 0.02 (s, 6H); ^13^C NMR (125 MHz, CDCl_3_) δ 154.9, 136.1, 116.7, 79.4, (75.2 + 74.5), (53.2 + 52.7), (49.0 + 48.6), (46.3 + 45.6), 35.6, 28.7, 25.9, 18.2, −4.4; ESMS *m/z* = 342 (M + H)^+^.

***tert*-Butyl 3-(*tert*-butyldimethylsilyloxy)-4-(oxiran-2-ylmethyl)-pyrrolidine-1-car­boxylate (10)**. A solution of **9** (181 mg, 0.53 mmol) in CH_2_Cl_2_ (10 mL) was cooled to 0 °C. *m*-CPBA (149 mg, 0.86 mmol, 77% pure, 1.3 equiv) was added, and the mixture was stirred for 40 h. 20% NaHSO_4_ solution (10 mL) was added, the mixture was stirred for 15 min, and the layers were separated. The organic layer was washed with NaHCO_3_ (2 × 10 mL), saturated NH_4_Cl (10 mL) and brine (10 mL), dried over Na_2_SO_4_ and concentrated *in vacuo.* The crude product was purified using flash column chromatography (silica gel, ethyl acetate/hexanes, 1:5) to afford **10** as an inseparable mixture of diastereomers (141 mg, 0.39 mmol, 74%). ^1^H NMR (500 MHz, CDCl_3_) δ 3.95 (m, 1H), 3.84 (m, 1H), 3.66–3.38 (m, 4H), 3.12–2.94 (m, 2H), 2.75 (dd, *J* = 4.5, 26.5 Hz, 1H), 2.25 (m, 1H), 1.71 (m, 1H), 1.45 (s, 9H), 0.88 (s, 9H), 0.07 (d, 6H); ^13^C NMR (125 MHz, CDCl_3_) δ 154.8, 79.6, (75.6 + 74.9), (53.0 + 52.5), (51.4 + 50.9), (49.3 + 48.9), 47.2, 45.1, (44.2 + 43.8), 28.8, 26.0, 18.2, −4.4; ESMS *m/z* = 358 (M + H)^+^, 380 (M + Na)^+^.

***tert*-Butyl 3-(3-bromo-2-hydroxypropyl)-4-(*tert*-butyldimethyl-silyloxy)pyrrolidine-1-carboxylate (11).** To a flame dried flask containing lithium bromide (55 mg, 0.62 mmol) under dry N_2_ was added a solution of **10** (141 mg, 0.39 mmol) in dry THF (5 mL). Acetic acid (57 μL, 1 mmol) was added dropwise, and the mixture was stirred for 16 h. NaHCO_3_ solution (10 mL) was added, and the product was extracted with ethyl acetate (3 × 10 mL). The organic layers were combined, dried over Na_2_SO_4_ and concentrated *in vacuo.* The crude product was purified using flash column chromatography (silica gel, ethyl acetate/hexanes, 1:4) to afford **11** as two diastereomers. The diastereomers could be separated, but were combined for further reactions (combined: 146 mg, 0.33 mmol, 85%). Diastereoisomer A: ^1^H NMR (500 MHz, CDCl_3_) δ 3.92 (m, 2H), 3.67 (m, 1H), 3.52 (m, 1H), 3.38 (m, 1H), 3.09–2.96 (m, 2H), 2.56 (m, 1H), 2.20–2.12 (m, 1H), 1.76–1.69 (m, 1H), 1.58–1.49 (m, 1H), 1.46 (s, 9H), 0.89 (s, 9H), 0.08 (m, 6H); ^13^C NMR (125 MHz, CDCl_3_) δ 154.8, 79.8, (76.2 + 75.6), 69.8, (53.1 + 52.5), (49.3 + 48.8), (43.5 + 42.8), 40.4, 36.8, 28.8, 26.1, 18.2, −4.3). Diastereoisomer B: ^1^H NMR (500 MHz, CDCl_3_) δ 3.94 (m, 1H), 3.83 (m, 1H), 3.67–3.58 (m, 1H), 3.52 (m, 1H), 3.41 (m, 1H), 3.11–2.96 (m, 2H), 2.54 + 2.47 (dd, *J* = 4.5, 38 Hz, 1H), 2.24 (m, 1H), 1.71 (m, 1H), 1.61 (s, 1H), 1.46 (s, 9H), 0.89 (s, 9H), 0.08 (m, 6H); ^13^C NMR (125 MHz, CDCl_3_) δ 154.8, 79.7, (76.2 + 75.3), 70.1, (52.7 + 52.3), (49.9 + 48.8), (43.8 + 43.1), 39.8, 36.8, 28.8, 26.0, 18.2, -4.3); ESMS *m/z* = 460, 462 (1:1) (M + Na)^+^, 897, 899, 901 (1:2:1) (2M + Na)^+^.

***tert*-Butyl 3-(3-bromo-2-oxopropyl)-4-(*tert*-butyldimethylsilyloxy)pyrrolidine-1-car‑boxylate (12).** A 3-necked flask equipped with stir bar and addition funnel was flame dried, sealed and allowed to cool under dry N_2_. Dry CH_2_Cl_2_ (15 mL) and DMSO (45 μL, 0.66 mmol) were added, and the mixture was cooled to −78 °C. Oxalyl chloride (250 μL, 0.5 mmol, 2M in CH_2_Cl_2_) was added, and the mixture was stirred for 5 min. A solution of **11** (146 mg, 0.33 mmol) in dry CH_2_Cl_2_ (5 mL) was added dropwise via the addition funnel. The mixture was stirred at −78 °C for 1 h. Triethylamine (91 μL, 0.63 mmol) was added, and the mixture was allowed to warm to room temperature. The reaction was quenched with brine (10 mL), and the product was extracted with CH_2_Cl_2_. The organic layers were combined, dried over Na_2_SO_4_ and concentrated *in vacuo.* The crude product was purified using flash column chromatography (silica gel, ethyl acetate/hexanes, 1:5) to afford **12** (84 mg, 0.19 mmol, 58%). ^1^H NMR (500 MHz, CDCl_3_) δ 4.07 (m, 1H), 3.90–3.86 (m, 2H), 3.72–3.46 (m, 2H), 3.10 (m, 1H), 2.98–2.77 (m, 2H), 2.61–2.44 (m, 2H), 1.45 (s, 9H), 0.87 (s, 9H), 0.06 (m, 6H); ^13^C NMR (125 MHz, CDCl_3_) δ 201.0, 154.8, 79.8, (75.0 + 74.4), (52.8 + 52.2), (49.1 + 48.7), 48.4, (42.3 + 42.1), (41.6 + 41.3), 34.3, 28.8, 26.0, 18.2, −4.4; ESMS *m/z* = 458, 460 (1:1) (M + Na)^+^, 893, 895, 897 (1:2:1) (2M + Na)^+^.

***tert*-Butyl 3-[(2-aminothiazol-4-yl)methyl]-4-(*tert*-butyldi-methylsilyloxy)­pyrroli­di­ne-1-carboxylate (13).** A solution of **12** (84 mg, 0.19 mmol) and thiourea (15 mg, 0.2 mmol) in ethanol (10 mL) was refluxed for 5 h. The mixture was poured into brine (20 mL) and extracted with ethyl acetate. The organic layers were combined, dried over Na_2_SO_4_ and concentrated *in vacuo.* The crude product was purified using flash column chromatography (silica gel, ethyl acetate/hexanes, 1:1) to afford **13** (62 mg, 0.15 mmol, 79%) as a white solid. ^1^H NMR (500 MHz, CDCl_3_) δ 6.06 (s, 1H), 5.52 (s, 2H), 3.98 (m, 1H), 3.62–3.50 (m, 2H), 3.18–3.04 (m, 2H), 2.62 (m, 1H), 2.35 (m, 1H), 1.45 (s, 9H), 0.86 (s, 6H), 0.02 (s, 6H); ^13^C NMR (125 MHz, CDCl_3_) δ 168.5, 155.1, 150.4, 103.3, 79.6, (75.4 + 74.6), (53.3 + 52.8), (49.3 + 48.9), (46.3 + 45.7), 33.3, 28.8, 26.1, 18.3, −4.5; ESMS *m/z* = 414 (M + H)^+^.

***tert*-Butyl 3-({2-[bis(*tert*-butoxycarbonyl)amino]thiazol-4-yl}-methyl)-4-(*tert*-butyl­dimethylsilyloxy)pyrrolidine-1-carboxylate (14).** To a solution of **13** (62 mg, 0.15 mmol) in dry THF (5 mL) were added Boc_2_O (82 mg, 0.37 mmol) and DMAP (10 mg). The mixture was stirred under N_2_ for 16 h. The solvent was removed *in vacuo*, and the crude product was purified using flash column chromatography (silica gel, ethyl acetate/hexanes, 1:6) to afford **14** (83 mg, 0.135 mmol, 91%) as a white solid. ^1^H NMR (500 MHz, CDCl_3_) δ 6.71 (s, 1H), 4.00 (m, 1H), 3.64–3.43 (m, 2H), 3.17–3.00 (m, 2H), 2.88–2.77 (m, 1H), 2.51–2.41 (m, 2H), 1.51 (s, 18H), 1.43 (s, 9H), 0.86 (s, 9H), 0.03 (s, 6H); ESMS *m/z* = 614 (M + H)^+^.

***tert*-Butyl 3-({2-[bis(*tert*-butoxycarbonyl)amino]thiazol-4-yl}-methyl)-4-hydroxy­pyrrolidine-1-carboxylate (15).** To a solution of **14** (375 mg, 0.61 mmol) in anhydrous THF (5 mL) was added TBAF (780 μL, 0.78 mmol, 1M solution in THF) dropwise, and the mixture was stirred overnight. The reaction mixture was poured into brine and extracted with ethyl acetate (3 × 25 mL). The organic layers were combined, dried over Na_2_SO_4_ and concentrated *in vacuo.* The crude product was purified using flash column chromatography (silica gel, ethyl acetate/hexanes, 1:1) to afford **15** (300 mg, 0.60 mmol, 98%) as a white solid. ^1^H NMR (500 MHz, CDCl_3_) δ 6.80 (s, 1H), 4.16 (m, 1H), 3.75–3.59 (m, 2H), 3.20 (m, 1H), 3.06 (m, 1H), 2.81–2.72 (m, 2H), 2.40–2.30 (m, 1H), 1.53 (s, 18H), 1.45 (s, 9H); ^13^C NMR (125 MHz, CDCl_3_) δ 158.9, 154.7, 150.6, 149.8, 112.7, 85.1, 79.5, (75.1 + 74.3), 64.5, (52.7 + 52.3), 49.6, (45.9 + 45.3), 33.4, 28.7, 27.9; ESMS *m/z* = 500 (M + H)^+^, 522 (M + Na)^+^.

**General procedure for the Mitsunobu reaction to form 16 and 28a–c.** To a solution of PPh_3_ (1.1 equiv) and phthalimide (1.1 equiv) in anhydrous THF (5mL) was added **15** or **7a–c** as a solution in anhydrous THF (5 mL). DIAD (1.1 equiv) was added dropwise, and the solution was stirred overnight. The reaction mixture was poured into saturated NaHCO_3_ (aq) and extracted with ethyl acetate (3 × 25 mL). The organic layers were combined, dried over Na_2_SO_4_ and concentrated *in vacuo.* The crude product was purified using flash column chromatography (silica gel, ethyl acetate/hexanes, 1:3) to afford **16** or **28a–c** as a white solid.

***tert*-Butyl 3-({2-[bis(*tert*-butoxycarbonyl)amino]thiazol-4-yl}-methyl)-4-(1,3-dioxoisoindolin-2-yl)pyrrolidine-1-carboxylate (16).** (333 mg, 0.53 mmol, 88%). ^1^H NMR (500 MHz, CDCl_3_) δ 7.86 (s, 2H), 7.77 (s, 2H), 6.65 (s, 1H), 4.98 (m, 1H), 3.91 (m, 2H), 3.63 (m, 1H), 3.43 (m, 1H), 3.08 (m, 1H), 2.78 (m, 1H), 2.55 (m, 1H), 1.52 (m, 27H); ^13^C NMR (125 MHz, CDCl_3_) δ 168.5, 158.4, 154.6, 150.2, 149.7, 134.5, 131.7, 123.6, 112.5, 84.6, 79.5, 64.5, (52.3 + 51.5), (49.8 + 49.1), (42.5 + 41.5), 30.5, 28.7, 27.9; ESMS *m/z* = 629 (M + H)^+^, 651 (M + Na)^+^.

**General procedure for the formation of 17 and 29a–c.** To a solution of **16** or **28a–c** in methanol (3 mL) was added 50% aqueous hydrazine (3 mL) dropwise. The solution was stirred at room temperature for 14 h. 2N HCl (15 mL) was added dropwise until the pH reached approximately 5, and the mixture was stirred a further 2 h. The solution was poured into saturated K_2_CO_3_ (20 mL, final pH ~10) and extracted with CH_2_Cl_2_ (5 × 15 mL). The organic layers were combined, dried over Na_2_SO_4_ and concentrated *in vacuo* to afford **17** or **29a–c** as a white solid.

***tert*-Butyl 3-amino-4-{[2-(*tert*-butoxycarbonylamino)-thiazol-4-yl]methyl}­pyrrolidine-1-carboxylate (17).** (76 mg, 0.19 mmol, 95%). Note: one of the Boc groups protecting the aminothiazole was removed during this procedure. ^1^H NMR (500 MHz, CDCl_3_) δ 6.53 (s, 1H), 3.57–3.40 (m, 2H), 3.36–3.16 (m, 2H), 2.86 (m, 1H), 2.75 (m, 1H), 2.64 (m, 1H), 2.46 (m, 1H), 1.54 (s, 9H), 1.44 (s, 9H); ^13^C NMR (125 MHz, CDCl_3_) δ 161.8, 160.6, 154.9, 152.7, 149.7, 107.6, 82.6, 79.5, (54.6 + 54.3), (52.7 + 51.8), (48.9 + 48.5), (43.6 + 43.0), 29.2, 28.7, 28.4; ESMS *m/z* = 399 (M + H)^+^.

**Ethyl 2-(4-chlorobenzylamino)acetate (18)**. Ethyl glycinate (700 mg, 5 mmol) and 4-chlorobenzyl chloride (480 mg, 3 mmol) were dissolved in methanol (10 mL). DIEA (872 μL, 5 mmol) was added, and the mixture was refluxed for 14 h. The solvent was removed *in vacuo* and the residue was purified using flash column chromatography (silica gel, ethyl acetate/methanol, 9:1) to afford **18** as a colorless oil (256 mg, ~1.15 mmol, 38%). A mixture of methyl and ethyl esters was formed. ^1^H NMR (500 MHz, CDCl_3_) δ 7.28 (s, 4H), 4.18 (q, *J* = 9 Hz, 2H), 3.77 (s, 2H), 3.38 (s, 2H), 1.94 (br, 1H), 1.27 (t, *J* = 9 Hz, 3H); ^13^C NMR (125 MHz, CDCl_3_) δ 172.5, 138.2, 133.0, 129.8, 128.7, 61.0, 52.8, 50.2, 14.5; ESMS *m/z* = 228/230 (3:1) (M + H)^+^.

**Ethyl 2-[*tert*-butoxycarbonyl(4-chlorobenzyl)amino]-acetate (19).** To a solution of **18** (256 mg, 1.15 mmol) in MeOH (10 mL) was added DIEA (280 μL, 1.5 mmol) and Boc_2_O (327 mg, 1.5 mmol). The mixture was stirred for 4 h. The solvent was removed *in vacuo*, and the crude residue was dissolved in sat NH_4_Cl solution. The product was extracted with ethyl acetate (3 × 15 mL). The organic layers were combined, dried over Na_2_SO_4_ and concentrated *in vacuo.* The crude product was purified using flash column chromatography (silica gel, ethyl acetate/hexanes, 1:4) to afford **19** as a white solid (406 mg, 1.14 mmol, 99%). ^1^H NMR (500 MHz, CDCl_3_) δ 7.28 (m, 2H), 7.12 (m, 2H), (4.50 + 4.47) (s, rotamers, 2H), 4.16 (m, 2H), 3.91 (s, 1H), 3.77 (s, 1H), 1.46 (s, 9H), 1.25 (t, *J* = 8.5 Hz, 3H); ^13^C NMR (125 MHz, CDCl_3_) δ 169.9, 155.7, (136.4 + 136.1), 133.4, 129.6, 128.9, 80.9, 61.3, (51.3 + 50.8), (48.6 + 48.2), 28.5, 14.4; ESMS *m/z* = 328/330 (3:1) (M + H)^+^.

**2-[*tert*-Butoxycarbonyl(4-chlorobenzyl)amino]acetic acid (20).** To a solution of **19** (406 mg, 1.14 mmol) in methanol (3 mL) was added 1 N NaOH (3 mL) dropwise. The mixture was stirred for 14 h. The mixture was acidified to pH 2 using 2N HCl, and the product was extracted with ethyl acetate (3 × 15 mL). The organic layers were combined, dried over Na_2_SO_4_, and concentrated *in vacuo* to afford **20** as a white solid (322 mg, 1.08 mmol, 95%). ^1^H NMR (500 MHz, CDCl_3_) δ 10.84 (br, 1H), 7.30 (m, 2H), 7.20 (m, 2H), 4.48 (d, rotamers, 2H), 3.97 (s, 1H), 3.82 (s, 1H), 1.47 (s, 9H); ^13^C NMR (125 MHz, CDCl_3_) δ 175.5, 155.9, 136.0, 133.6, 129.7, 129.0, 81.5, (51.4 + 50.7), 48.1, 28.5; ESMS (-ve mode) *m/z* = 298/300 (3:1) (M − H)^-^.

***tert*-Butyl 4-chlorobenzyl{2-[methoxy(methyl)amino]-2-oxoethyl}carbamate (21).** To a solution of **20** (322 mg, 1.1 mmol) in dry CH_2_Cl_2_ (5 mL) was added DIEA (262 μL, 1.5 mmol). The solution was cooled to 0 °C and EDC (288 mg, 1.5 mmol) and HOBt (203 mg, 1.5 mmol) were added. After 5 min, HN(OMe)Me.HCl (147 mg, 1.5 mmol) and DIEA (262 μL, 1.5 mmol) were added. The mixture was stirred at room temperature for 14 h. The mixture was diluted with CH_2_Cl_2_ (15 mL) and washed with 1N HCl (2 × 20 mL), sat NaHCO_3_ (2 × 20 mL) and brine (1 × 20 mL), dried over Na_2_SO_4_ and concentrated *in vacuo*. The crude product was purified using flash column chromatography (silica gel, ethyl acetate/hexanes, 1:3) to afford **21** as a white solid (360 mg, 1.05 mmol, 97%). ^1^H NMR (500 MHz, CDCl_3_) δ 7.31–7.18 (m, 4H), (4.54 + 4.50) (s, rotamers, 2H), 4.10 (s, 1H), 3.96 (s, 1H), (3.66 + 3.62) (s, rotamers, 3H), 3.18 (s, 3H), 1.46 (s, 9H); ^13^C NMR (125 MHz, CDCl_3_) δ 169.9, 156.1, 136.8, 133.1, 129.6, 128.9, 80.6, 61.5, (51.3 + 50.6), 47.3, 32.6, 28.6; ESMS *m/z* = 343/345 (3:1) (M + H)^+^.

***tert*-Butyl 4-chlorobenzyl(2-oxoethyl)carbamate (22)**. A solution of **21** (137 mg, 0.4 mmol) in anhydrous THF (3 mL) was cooled to 0 °C. A solution of lithium aluminum hydride (0.5 mL, 0.5 mmol, 1 M in THF) was added dropwise, and the mixture was stirred at 0 °C for 1 h. The reaction was quenched by the addition of 20% sodium bisulfate solution (15 mL). The product was extracted with ethyl acetate (2 × 15 mL) and the combined organic layers were washed with 1N HCl (2 × 20 mL), saturated NaHCO_3_ (2 × 20 mL) and brine (1 × 20 mL), dried over Na_2_SO_4_ and concentrated *in vacuo*. The crude product was purified using flash column chromatography (silica gel, ethyl acetate/hexanes, 1:3) to afford **22** as a white solid (108 mg, 0.38 mmol, 94%). ^1^H NMR (500 MHz, CDCl_3_) δ (9.51 + 9.44) (s, rotamers, 1H), 7.31–7.15 (m, 4H), (4.50 + 4.56) (s, rotamers, 2H), 3.95 (s, 1H), 3.80 (s, 1H), 1.48 (9H); ^13^C NMR (125 MHz, CDCl_3_) δ 198.4, 155.5, 136.0, 133.7, 129.6, 129.1, 81.5, 56.8, (51.7 + 51.2), 28.5.

**General reductive amination procedure to form 23 and 31a–c.** To a solution of **17** or **28a–c** in methanol (3 mL) was added a solution of **22** (1.0 equiv) in CH_2_Cl_2_ (1 mL). The mixture was stirred for 15 min at room temperature, then NaHB(OAc)_3_ (1.1 equiv) was added. The mixture was stirred for 90 min then poured into NaHCO_3_ solution (15 mL). The product was extracted with EtOAc (3 × 15 mL), dried over Na_2_SO_4_ and concentrated *in vacuo*. The crude product was purified using flash column chromatography (silica gel, ethyl acetate/hexanes, 3:1) to afford **17** or **28a–c** as an oily solid.

***tert*-Butyl 3-{2-[*tert*-butoxycarbonyl(4-chloro-benzyl)amino]ethylamino}-4-{[2-(*tert*-butoxy-carbonylamino)thiazol-4-yl]methyl}pyrrolidine-1-carboxylate (17).** (100 mg, 0.15 mmol, 88%) as a white solid. ^1^H NMR (500 MHz, CDCl_3_) δ 7.28 (s, 2H), 7.17 (s, 2H), 6.51 (s, 1H), 4.47 (m, 2H), 3.48–3.06 (m, 6H), 2.77–2.62 (m, 4H), 2.45 (m, 1H), 2.01 (m, 1H), 1.54–1.44 (m, 27H); ^13^C NMR (125 MHz, CDCl_3_) δ 159.9, 155.0, 152.9, 149.9, 133.2, 128.9, 108.1, 82.4, 80.6, 79.4, 58.6, 53.9, 51.3, 50.9, 49.7, 49.4, 47.1, 29.2, 28.8, 28.5; ESMS *m/z* = 666/668 (3:1) (M + H)^+^.

**General procedure for removal of Boc groups to afford 3 or 4a–c.** A solution of HCl in dioxanes (4 N, 3mL) was added to **23** or **31a–c**, and the mixture was stirred overnight. The deprotection was monitored by removing small aliquots, quenching and analyzing by ESMS. Once the deprotection was complete, the excess solvent and HCl were removed under a stream of N_2_. The residue was dissolved in H_2_O (10 mL) and washed with ethyl acetate (2 × 10 mL), and the water was removed. The residue was dissolved in a minimum amount of methanol and precipitated with anhydrous ether. The ether was decanted, and the white solids were dried under vacuum to give **3** or **4a–c** as the tetrahydrochloride salt.

***N*^1^-{4-[(2-Aminothiazol-4-yl)methyl]-pyrrolidin-3-yl}-*N*^2^-(4-chlorobenzyl)ethane-1,2-di-amine (3)**. (42 mg, 0.083 mmol, 55%): mp 175–177 °C; ^1^H NMR (500 MHz, D_2_O) δ 7.32 (s, 4H), 6.50 (s, 1H), 4.16 (m, 3H), 3.78 (m, 1H), 3.65–3.37 (m, 6H), 3.23 (m, 1H), 3.01 (m, 1H), 2.93–2.89 (m, 1H), 2.65 (m, 1H); ESMS *m/z* = 366/368 (3:1) (M + H)^+^;.HRMS (ESMS) calcd for C_17_H_24_ClN_5_S: 366.15192, 368.14897, found: 366.15150 (M + H, ^35^Cl); 368.14924 (M + H, ^37^Cl).

**Ethyl 2-chloro-3-methyloxirane-2-carboxylate (30a).** A fresh solution of sodium ethoxide was prepared by addition of small pieces of sodium metal (0.3 g, 13 mmol) to ethanol (5 mL) at 0 °C. Once the sodium had reacted, the solution was added via cannula to a solution of ethyl dichloroacetate (1.6 mL, 12.7 mmol) and acetaldehyde (840 μL, 15 mmol) in anhydrous ether (10 mL) at 0 °C. The mixture was stirred at 0 °C for 1 h. Ether (10 mL) and saturated NH_4_Cl (10 mL) were added to the mixture and the layers were separated. The aqueous layer was extracted with ether (2 × 10 mL). The organic layers were combined, dried over MgSO_4_ and concentrated to a colorless oil. A large portion of the product was lost when put under reduced pressure to remove solvent; therefore, no accurate yield was obtained. When sodium methoxide was used as the base, the major product was the methyl ester. Mixture of *cis* and *trans* stereoisomers. Stereoisomer A: ^1^H NMR (500 MHz, CDCl_3_) δ 4.04 (s, 3H), 3.74 (q, *J* = 8 Hz, 1H), 1.56 (d, *J* = 7 Hz, 3H); Stereoisomer B: ^1^H NMR (500 MHz, CDCl_3_) δ 4.04 (s, 3H), 3.54 (q, *J* = 6 Hz, 1H), 1.46 (d, *J* = 7 Hz, 3H).

**Methyl 2-chloro-3-isopropyloxirane-2-carboxylate (30b).** The procedure used to create **30a** was repeated, except that isobutyraldehyde was used instead of acetaldehyde. As sodium methoxide was used as the base, a mixture of the methyl and ethyl esters of **30b** were formed, with the methyl ester being the major product (9.0 mmol, 71%). ^1^H NMR (500 MHz, CDCl_3_) δ 3.83 (s, 3H), 3.08 (d, *J* = 11 Hz, 1H), 1.88 (m, 1H), 1.17 (d, *J* = 8.5 Hz, 3H), 1.06 (d, *J* = 8 Hz, 3H).

***tert*-Butyl 3-(cyanomethyl)-4-hydroxypyrrolidine-1-carboxylate (24).** A 3-necked flask equipped with stir bar and addition funnel was flame dried, sealed and allowed to cool under a dry N_2_ atmosphere. The flask was charged with dry THF (10 mL) and diisopropylamine (280 μL, 2 mmol), and the mixture was cooled to −78 °C. *n*-BuLi (2.3 mL, 1.4 M in hexanes, 1.8 mmol) was added dropwise via the addition funnel. The mixture was allowed to warm to room temperature and stirred for 30 min, before being cooled down to −78 °C. Anhydrous acetonitrile (104 μL, 2 mmol) was added dropwise, and the mixture was allowed to warm to room temperature. After 15 min of stirring the mixture was cooled to −5 °C. A solution of **5** (370 mg, 2 mmol) in anhydrous THF (10 mL) was added dropwise. The mixture was stirred for 2 h then quenched with saturated NH_4_Cl (aq.). The product was extracted with ethyl acetate (3 × 15 mL), dried over Na_2_SO_4_ and concentrated. The crude product was purified using flash column chromatography (silica gel, ethyl acetate/hexanes, 3:1) to afford **24** as a pale yellow oil (294 mg, 1.30 mmol, 72%). ^1^H NMR (500 MHz, CDCl_3_) δ 4.14 (m, 1H), 3.70 (m, 2H), 3.24 (m, 2H), 2.53–2.41 (m, 3H), 1.46 (s, 9H); ^13^C NMR (125 MHz, CDCl_3_) δ 154.7, 118.0, 80.4, (73.5 + 72.8), (52.4 + 52.0), (48.6 + 48.2), (42.5 + 41.9), 28.7, 18.7; ESMS *m/z* = 249 (M + Na)^+^, 475 (2M + Na)^+^.

***tert*-Butyl 3-(2-amino-2-thioxoethyl)-4-hydroxypyrrolidine-1-carboxylate** **(25)**. To a solution of **24** (226 mg, 1 mmol) in ethanol (3 mL) was added 50% (NH_4_)_2_S (aq., 0.3 mL, 4.5 mmol). The mixture was stirred for 44 h. The mixture was added to saturated NaCl solution (15 mL) and extracted with ethyl acetate (3 × 15 mL). The organic layers were combined, dried over Na_2_SO_4_ and concentrated *in vacuo*. The crude product was purified using flash column chromatography (silica gel, ethyl acetate/hexanes, 9:1) to afford **25** as a colorless, viscous oil (0.34 mmol, 34%). Unreacted **24** was also recovered and could be submitted to the same conditions to generate more thioamide. ^1^H NMR (500 MHz, CDCl_3_) δ 8.82 (br, 2H), 4.13 (m, 1H), 3.66 (m, 2H), 3.25 (m, 2H), 2.89–2.65 (m, 3H), 1.50 (s, 9H); ^13^C NMR (125 MHz, CDCl_3_) δ 206.3, 154.5, 78.8, (74.0 + 73.4), (52.9 + 52.6), (49.2 + 48.9), 46.3, 45.6, 28.2; ESMS *m/z* = 261 (M + H)^+^, 283 (M + Na)^+^.

**General procedure for synthesis of 26a–c.**

To a solution of **25** in ethanol (10 mL) was added ethylbromopyruvate, **30a** or **30b** (1.1 equiv). The mixture was refluxed for 5 h. The solution was cooled to room temperature and neutralized with diisopropylethylamine (1.25 equiv). Boc_2_O (1.25 equiv) was added and the mixture was stirred overnight. The solvent was removed *in vacuo*, and the residue was dissolved in brine (15 mL) and extracted with ethyl acetate (3 × 15 mL). The organic layers were combined, dried over Na_2_SO_4_ and concentrated *in vacuo*. The crude product was purified using flash column chromatography (silica gel, ethyl acetate/hexanes, 1:1) to afford **26a–c** as a white solid.

**Ethyl 2-{[1-(*tert*-butoxycarbonyl)-4-hydroxypyrrolidin-3-yl]methyl}thiazole-4-car­boxylate (26a).** (68 mg, 0.19 mmol, 85%). ^1^H NMR (500 MHz, CDCl_3_) δ 8.06 (s, 1H), 4.38 (q, *J* = 7 Hz, 2 H), 4.22 (m, 1H), 3.72 (m, 2H), 3.26–3.09 (m, 4H), 2.56 (m, 1H), 1.43 (s, 9H), 1.37 (t, *J* = 6.5 Hz, 3H); ^13^C NMR (125 MHz, CDCl_3_) δ 169.6, 161.2, 154.7, 146.9, 127.4, 79.8, (75.0 + 74.3), 61.8, 52.7, (49.7 + 49.5), (45.6 + 44.7), 35.4, 28.7, 14.5; ESMS *m/z* = 357 (M + H)^+^.

**Ethyl 2-{[1-(*tert*-butoxycarbonyl)-4-hydroxypyrrolidin-3-yl]methyl}-5-methyl­thia­zole-4-carboxylate (26b).** (196 mg, 0.55 mmol, 79%). ^1^H NMR (500 MHz, CDCl_3_) δ 4.15 (m, 1H), 3.92 (s, 3H), 3.71 (m, 2H), 3.23 (m, 1H), 3.08 (m, 2H), 2.74 (s, 3H), 2.83–2.45 (m, 2H), 1.45 (s, 9H); ^13^C NMR (125 MHz, CDCl_3_) δ 207.7, 164.8, 162.7, 154.9, 145.3, 140.3, 94.6, 80.0, (74.8 + 74.1), (52.9 + 52.7), 52.5, (49.7 + 49.1), (46.6 + 45.5), 35.0, 28.7, 13.3; ESMS *m/z* = 357 (M + H)^+^.

**Ethyl 2-{[1-(*tert*-butoxycarbonyl)-4-hydroxypyrrolidin-3-yl]methyl}-5-isopropyl­thiazole-4-carboxylate (26c).** (614 mg, 1.06 mmol, 76%). ^1^H NMR (500 MHz, CDCl_3_) δ 4.07 (m, 2H), 3.82 (s, 3H), 3.64 (m, 2H), 3.17 (m, 1H), 3.09–2.92 (m, 3 H), 1.36 (s, 9H), 1.22 (d, *J* = 8.5 Hz, 6H); ^13^C NMR (125 MHz, CDCl_3_) δ 164.7, 162.4, 159.3, 154.5, 138.5, 94.6, 79.7, (75.0 + 74.3), (53.0 + 52.5), 52.4, (49.9 + 49.5), (45.3 + 44.5), 35.3, 28.7, 28.0, 25.3; ESMS *m/z* = 385 (M + H)^+^.

**General Procedure for formation of 27a–c** To a solution of **26** in methanol (3 mL) was added 1 N NaOH (3 mL) dropwise. The mixture was stirred for 14 h. The mixture was acidified to pH 3 using 2N HCl, and the product was extracted with ethyl acetate (3 × 15 mL). The organic layers were combined, dried over Na_2_SO_4_ and concentrated *in vacuo* to afford **27** as a white solid

**2-{[1-(*tert*-Butoxycarbonyl)-4-hydroxypyrrolidin-3-yl]methyl}thiazole-4-carboxylic acid (27a)**. (59 mg, 0.18 mmol, 95%). ^1^H NMR (500 MHz, CDCl_3_) δ 8.16 (s, 1H), 4.25 (m, 1H), 3.70 (m, 2H), 3.32–3.17 (m, 4H), 2.64 (m, 1H), 1.45 (s, 9H); ^13^C NMR (125 MHz, CDCl_3_) δ 169.7, 163.3, 155.0, 146.5, 128.5, 80.3, (74.5 + 73.8), (52.6 + 52.3), (49.6 + 49.2), (45.7 + 44.9), 34.9, 28.7; ESMS (-ve mode) *m/z* = 327 (M − H)^-^.

**2-{[1-(*tert*-Butoxycarbonyl)-4-hydroxypyrrolidin-3-yl]methyl}-5-methylthiazole-4-carboxylic acid (27b).** (0.55 mmol, quant) ^1^H NMR (500 MHz, CDCl_3_) δ 4.20 (m, 1H), 3.71 (m, 2H), 3.25 (m, 1H), 3.11 (m, 2H), 2.73 (s, 3H), 2.55 (m, 2H), 1.45 (s, 9H); ^13^C NMR (125 MHz, CDCl_3_) δ 175.5, 164.4, 154.9, 145.6, 140.6, 80.1, (74.5 + 73.8), (52.6 + 52.3), (49.6 + 49.3), (45.4 + 44.6), 34.6, 28.7, 13.3. ESMS *m/z* = 343 (M + H)^+^, 343 (M + Na)^+^, 685 (2M + H)^+^, 685 (2M + Na)^+^.

**2-{[1-(*tert*-Butoxycarbonyl)-4-hydroxypyrrolidin-3-yl]methyl}-5-isopropylthiazole-4-carboxylic acid (27c).** (1.05 mmol, quant) ^1^H NMR (500 MHz, CDCl_3_) δ 4.30 (m, 1H), 4.14 (m, 1H), 3.68 (m, 2H), 3.36–2.99 (m, 4H), 2.55 (m, 1H), 1.46 (s, 9H), 1.32 (d, *J* = 8 Hz, 6H); ESMS *m/z* = 369 (M − H)^-^.

**General Procedure for formation of 7a–c via Curtius rearrangement** A 3-necked flask with stir bar, condenser, and 3 Å molecular sieves was flame dried under vacuum and allowed to cool under a dry nitrogen atmosphere. A solution of **27** in warm anhydrous *t*-BuOH (20 mL) was added via cannula, followed by triethylamine (1.1 equiv). The mixture was refluxed for 30 min then allowed to cool. Diphenylphosphoryl azide (1.02 equiv) was added, and the mixture was stirred at 50 °C for 30 min. The system was heated to reflux for 14 h. The sieves were removed by filtration and the solvent was removed *in vacuo*. The crude product was purified using flash column chromatography (silica gel, ethyl acetate/hexanes, 1:1) to afford **7a–c** as a white solid.

***tert*-Butyl 3-{[4-(*tert*-butoxycarbonylamino)thiazol-2-yl]methyl}-4-hydroxypyrrolidi­ne-1-carboxylate (7a).** (260 mg, 0.65 mmol, 53%). ^1^H NMR (500 MHz, CDCl_3_) δ 8.26 (br, 1H), 7.11 (s, 1H), 4.90 (m, 1H), 4.17 (m, 1H), 3.72 (m, 2H), 3.28 (m, 1H), 3.15–3.02 (m, 3H), 2.51 (m, 1H), 1.51 b(s, 9H), 1.45 (s, 9H); ^13^C NMR (125 MHz, CDCl_3_) δ 166.2, 154.6, 152,7, 147.5, 97.9, 81.1, 79.8, (75.0 + 74.3), (52.8 + 52.4), (49.7 + 49.4), (45.6 + 44.9), 34.9, 28.8, 28.6; ESMS *m/z* = 400 (M + H)^+^.

***tert*-Butyl 3-{[4-(*tert*-butoxycarbonylamino)-5-methylthiazol-2-yl]methyl}-4-hydro­xypyrrolidine-1-carboxylate (7b).** ^1^H NMR (500 MHz, CDCl_3_) δ 6.73 (s, 1H), 4.18 (m, 1H), 3.72 (m, 2H), 3.23 (m, 1H), 3.07–2.89 (m, 2H), 2.48 (m, 2H), 2.29 (s, 3H), 1.49 (s, 9H), 1.45 (s, 9H); ^13^C NMR (125 MHz, CDCl_3_) δ 163.2, 153.4, 142.4, 130.1, 81.1, 79.8, (75.1 + 74.5), (53.0 + 52.5), (49.9 + 49.6), (45.3 + 44.5), 35.4, 28.8, 28.5, 11.4; ESMS *m/z* = 414 (M + H)^+^, 436 (M + Na)^+^, 827 (2M + H)^+^, 849 (2M + Na)^+^.

***tert*-Butyl 3-{[4-(*tert*-butoxycarbonylamino)-5-isopropylthiazol-2-yl]methyl}-4-hy­droxypyrrolidine-1-carboxylate (7c).** ^1^H NMR (500 MHz, CDCl_3_) δ 6.92 (m, 1H), 4.12 (m, 1H), 3.71 (m, 2H), 3.21 (m, 2H), 3.07 (m, 1H), 2.98 (m, 2H), 2.48 (m, 1H), 1.48 (s, 9H), 1.45 (s, 9H), 1.25 (d, 6H); ^13^C NMR (125 MHz, CDCl_3_) δ 162.9, 154.5, 153.9, 139.7, 137.0, 94.6, 80.8, 79.6, (74.8 + 74.2), (52.8 + 52.4), (45.4 + 44.6), 35.5, 31.1, 28.7, 28.5, 27.0, 24.8. ESMS *m/z* = 442 (M + H)^+^, 464 (M + Na)^+^.

***tert*-Butyl 3-{[4-(*tert*-butoxycarbonylamino)thiazol-2-yl]methyl}-4-(1,3-dioxoiso­indolin-2-yl)pyrrolidine-1-carboxylate (28a).** (327 mg, 0.62 mmol, 95%). ^1^H NMR (500 MHz, CDCl_3_) δ 8.04 (br, 1H), 7.82 (d, *J* = 3 Hz, 2H), 7.74 (d, *J* = 3 Hz, 2H), 7.01 (br, 1H), 5.00 (m, 1H), 3.88–3.69 (m, 3H), 3.50 (m, 1H), 3.13–2.99 (m, 2H), 2.85 (m, 1H), 1.50 (s, 18H); ^13^C NMR (125 MHz, CDCl_3_) δ 168.3, 165.1, 154.2, 152.5, 147.5, 134.4, 131.6, 123.5, 97.6, 80.8, 79.6, (53.8 + 51.9), (51.0 + 50.6), (49.7 + 49.3), (42.8 + 42.0), 32.0, 28.7, 28.5; ESMS *m/z* = 551 (M + Na)^+^.

***tert*-Butyl 3-{[4-(*tert*-butoxycarbonylamino)-5-methylthiazol-2-yl]methyl}-4-(1,3-di­oxoisoindolin-2-yl)pyrrol-idine-1-carboxylate (28b).** ^1^H NMR (500 MHz, CDCl_3_) δ 7.84 (s, 2H), 7.76 (s, 2H), (6.96 + 6.87) (s, rotamers, 1H), 4.98 (m, 1H), 3.88–3.71 (m, 3H), 3.46 (m, 1H), 3.10–2.74 (m, 3H), 2.25 (s, 3H), 1.50 (s, 9H), 1.48 (s, 9H); ^13^C NMR (125 MHz, CDCl_3_) δ 168.4, 161.7, 154.5, 154.2, 142.3, 134.5, 131.7, 123.6, 80.5, 79.6, (51.9 + 51.1), 50.5, (49.7 + 49.3), (42.7 + 42.0), 32.3, 28.7, 28.4, 11.5; ESMS *m/z* = 565 (M + Na)^+^.

***tert*-Butyl 3-{[4-(*tert*-butoxycarbonylamino)-5-isopropylthiazol-2-yl]methyl}-4-(1,3-dioxoisoindolin-2-yl)pyrrolidine-1-carboxylate (28c).** ^1^H NMR (500 MHz, CDCl_3_) δ 7.83 (m, 2H), 7.76 (m, 2H), 6.54 (m, 1H), 4.99 (m, 1H), 3.88–3.74 (m, 3H), 3.49 (t, 1H), 3.23–3.05 (m, 2H), 2.92 (m, 1H), 2.80 (m, 1H), 1.47 (m, 18H), 1.20 (m, 6H); ^13^C NMR (125 MHz, CDCl_3_) δ 168.3, 161.5, 154.5, 154.2, 153.7, 139.9, 137.5, 134.4, 131.7, 123.5, 80.5, 79.7, 51.8, 51.1, 51.0, 50.6, 49.7, 49.3, (42.6 + 41.9), 32.5, 28.7, 28.5, 27.0, 24.8; ESMS *m/z* = 571 (M + H)^+^, 593 (M + Na)^+^.

***tert*-Butyl 3-amino-4-{[4-(*tert*-butoxycarbonylamino)-thiazol-2-yl]methyl}pyrroli­dine-1-carboxylate (29a).** (235 mg, 0.59 mmol, 95%). ^1^H NMR (500 MHz, CDCl_3_) δ 8.22 (s, 1H), 7.02 (s, 1H), 3.52 (m, 1H), 3.40 (m, 2H), 3.23–2.88 (m, 4H), 2.45 (m, 1H), 1.45 (s, 9H), 1.37 (s, 9H); ^13^C NMR (125 MHz, CDCl_3_) δ 166.2, 154.7, 152.7, 147.6, 97.6, 80.9, 79.5, (55.0 + 54.6), (52.5 + 51.5), (48.7 + 48.3), (44.2 + 43.5), 31.0, 28.7, 28.6; ESMS *m/z* = 399 (M + H)^+^.

***tert*-Butyl 3-amino-4-{[4-(*tert*-butoxycarbonylamino)-5-methylthiazol-2-yl]methyl}­pyrrolidine-1-carboxylate (29b).** ^1^H NMR (500 MHz, CDCl_3_) δ 7.01 (s, 1H), 3.44 (m, 2H), 3.20–2.99 (m, 3H), 2.83 (m, 1H), 2.43 (m, 2H), 2.21 (s, 3H), 1.41 (s, 9H), 1.37 (s, 9H); ^13^C NMR (125 MHz, CDCl_3_) δ 162.8, 154.8, 153.6, 142.3, 122.7, 80.6, 79.4, 54.8, (52.4 + 51.5), (48.7 + 48.3), (43.8 + 43.1), 31.2, 28.7, 28.4, 11.5; ESMS *m/z* = 413 (M + H)^+^.

***tert*-Butyl 3-amino-4-{[4-(*tert*-butoxycarbonylamino)-5-isopropylthiazol-2-yl]­methyl}pyrrolidine-1-carboxylate (29c).** ^1^H NMR (500 MHz, CDCl_3_) δ 6.76 (s, 1H), 3.73–3.46 (m, 3H), 3.29–3.07 (m, 4H), 2.93 (m, 1H), 2.54 (m, 1H), 1.48 (s, 9H), 1.45 (s, 9H), 1.26 (d, *J* = 5 Hz, 6H); ^13^C NMR (125 MHz, CDCl_3_) δ 162.6, 154.7, 153.9, 139.9, 137.6, 80.5, 79.5, (55.0 + 54.6), (52.5 + 51.5), (48.8 + 48.3), (43.8 + 43.1), 31.6, 28.7, 28.5, 27.1, 24.9; ESMS *m/z* = 441 (M + H)^+^, 881 (2M + H)^+^.

***tert*-Butyl 3-{2-[*tert*-butoxycarbonyl(4-chlorobenzyl)amino]ethylamino}-4-{[4-(*tert*-butoxycarbonylamino)thiazol-2-yl]methyl}pyrrolidine-1-carboxylate (31a).** (60 mg, 0.09 mmol, 38%). ^1^H NMR (500 MHz, CDCl_3_) δ 7.91 (br, 1H), 7.33–7.08 (m, 5H), 4.45 (m, 2H), 3.46–3.10 (m, 8H), 2.88–2.77 (m, 2H), 2.58 (m, 2H), 1.45 (m, 27H); ESMS *m/z* = 666/668 (3:1) (M + H)^+^.

***tert*-Butyl 3-{2-[*tert*-butoxycarbonyl(4-chlorobenzyl)amino]ethylamino}-4-{[4-(*tert*-butoxycarbonylamino)-5-methyl-thiazol-2-yl]methyl}pyrrolidine-1-carboxylate (31b).** ^1^H NMR (500 MHz, CDCl_3_) δ 7.29 (m, 2H), 7.17 (m, 2H), 6.86 (br, 1H), 4.49 (m, 2H), 3.53–3.04 (m, 8H), 2.80 (m, 2H), 2.57 (m, 2H), 2.90 (m, 3H), 1,46 (m, 27H); ^13^C NMR (125 MHz, CDCl_3_) δ 163.2, 154.9, 153.6, 142.1, 137.0, 133.2, 129.3, 128.9, 122.9, 80.6, 80.4, 79.5; ESMS *m/z* = 680/682 (3:1) (M + H)^+^.

***tert*-Butyl 3-{2-[*tert*-butoxycarbonyl(4-chlorobenzyl)amino]ethylamino}-4-{[4-(*tert*-butoxycarbonylamino)-5-isopropylthiazol-2-yl]methyl}-pyrrolidine-1-carboxylate (31c).** ^1^H NMR (500 MHz, CDCl_3_) δ 7.30 (m, 2H), 7.17 (m, 2H), 6.46 (m, 1H), 4.42 (m, 2H), 3.52–3.04 (m, 9H), 2.82 (m, 2H), 2.61 (m, 2H), 1.47 (m, 27H), 1.25 (m, 6H); ESMS *m/z* = 708/710 (3:1) (M + H)^+^, 730/732 (3:1) (M + Na)^+^.

**2-({4-[2-(4-Chlorobenzylamino)ethylamino]-pyrrolidin-3-yl}methyl)-5-methylthiazol-4(5*H*)-one (4b, actual structure in water).** ^1^H NMR (500 MHz, D_2_O) δ 7.29 (d, *J* = 9 Hz, 2H), 7.21 (d, *J* = 9Hz, 2H), 4.53 (t, *J* = 6.5 Hz, 1H), 4.41 (q, *J* = 9.5 Hz, 2H), 4.00 (t, *J* = 9.5 Hz, 2H), 3.76 (m, 3H), 3.62 (m, 2H), 3.36 (m, 2H), 3.16 (m, 1H), 3.10 (m, 1H), 2.73 (d, *J* = 19 Hz, 1H), 1.44 (d, *J* = 7 Hz, 3H); ^13^C NMR (125 MHz, CDCl_3_) δ 174.4, 173.4, 134.3, 131.5, 130.5, 129.2, 62.5, 54.5, 51.3, 50.4, 47.9, 43.9, 42.3, 34.3, 29.6, 20.8; ESMS *m/z* = 276/278 (3:1) unknown decomposition product.

**AutoDock Analysis.** AutoDock 3.0.5 was employed to perform the docking calculations [1]. For the protein structure (PDB code 1P6I), polar hydrogen atoms were added, and Kollman united atom charges were assigned [2]. Hydrogens were also added to the heme and H_4_B, and charges were calculated by the Gasteiger–Marsili method [3]. The charge of the Fe atom bound to heme was assigned +3. The nonpolar hydrogen atoms of heme and H_4_B were removed manually, and their charges were united with the bonded carbon atoms. Atomic solvation parameters and fragmental volumes were assigned using the AddSol utility. The 3D structures of the ligands were built and partial atomic charges were also calculated using the Gasteiger–Marsili method. The rotatable bonds in the ligands were defined using another AutoDock 3.0 auxiliary program, AutoTors, which also unites the nonpolar hydrogens and partial atomic charges to the bonded carbon atoms. The grid maps were calculated using AutoGrid. The dimensions of the grid box was 27 × 26 × 31 Å, and the grid spacing was set to 0.375 Å. Docking was performed using the Lamarckian genetic algorithm (LGA), and the pseudo-Solis and Wets method were applied for the local search. The procedure in detail used was that previously described [4,5].

**In vitro enzyme assay**. The NOS isoforms used were recombinant enzymes overexpressed in *E. coli.*  Rat nNOS [6], bovine eNOS [7], and murine macrophage iNOS [8], and were overexpressed and isolated as reported. The formation of nitric oxide was monitored using a hemoglobin capture assay as described previously. Briefly, a solution of nNOS or eNOS containing 10 μM L-arginine, 1.6 mM CaCl_2_, 11.6 μg/mL calmodulin, 100 μM DTT, 100 μM NADPH, 6.5 μM H_4_B, 125 μg/mL oxyhemoglobin, and varying concentrations of inhibitor in 100 mM Hepes (pH 7.4) was monitored at 30 °C. For the determination of inhibition of iNOS, no additional Ca^2+^ or calmodulin were added. The assay was initiated by the addition of enzyme, and the absorption of UV light at 400 nm was recorded over one minute. As NO was evolved and coordinated to the hemoglobin, the absorption at 400 nm increased, producing a value for the enzyme velocity under these conditions. A value for the initial rate was obtained when no inhibitor was added (*v_0_*). The velocity of the enzyme (*v*) was then determined in the presence of varying concentrations of inhibitor, until a concentration of inhibitor that reduced the enzyme velocity to half its initial value (*v/v_0_* ~ 0.5) was discovered. Concentrations of inhibitor above and below this value were tested and a graph of *v/v_0_* versus inhibitor concentration ([I]) was plotted. Experiments were repeated at least three times and until a R^2^ >0.99 was obtained. Extrapolation of this graph allowed the determination of an IC_50_ value. The K_i_ can be estimated from the IC_50_ if the K_m_ for the substrate is known, using the equations below. The K_m_ values used were: 1.3 μM (nNOS), 8.3 μM (iNOS) and 1.7 μM (eNOS).

% inhibition = 100 [I] / ([I] + K_i_ {1 + [S] / K_m_})

K_i_ = IC_50_ / (1 + [S] / K_m_)

**HPLC Analysis of 3**

Chromatographic conditions 1

Column: Symmetry C18, 150 × 4.6mm, 3.5 μm particle size

Instrument: PE LC-250B with PE Nelson 900 A/D detector

Mobile Phase: A – 0.1% TFA, B – acetonitrile

Flow rate: 1.0 ml/min

UV detection: 220 nm

Runtime: 70 min

Detector time: 70 min

Inj. volume: 10 μL

Diluent: Mobile phase A

Gradient (Time/%B): 0/5, 20/20, 50/95, 55/95, 60/5, 70/5

Compound **3**. Retention time 24.9 min, 98%

Chromatographic conditions 2

Column: Symmetry C18, 75 × 4.6mm, 3.5 μm particle size

Instrument: Quaternary LC Pump Model 200Q/410 with LC785A detector

Mobile Phase: A – 0.1% TFA, B – MeOH

Flow rate: 1.5 ml/min

UV detection: 254 nm

Runtime: 10 min

Detector time: 10 min

Inj. volume: 10 μL

Diluent: Mobile phase A

Gradient (Time/%B): 0/10, 1/10, 4/75, 7/75, 7.1/10, 10/10

Compound **3**. Retention time 4.35 min, 98%

## References

Morris, G. M. et al. *J. Comp. Chem.* **1998**, *19,* 1639-1662.

Weiner, S. J.; Kollman, P. A.; Case, D. A.; Singh, U. C.; Ghio, C.; Alagona, G.; Profeta, S.; Weiner, P. A. *J. Am. Chem. Soc.* **1984**, *106*, 765-784.

Gasteiger, J.; Marsili, M. *Tetrahedron* **1980**, 36, 3210-3328.

Ji, H.; Stanton, B. Z.; Igarashi, J.; Li, H.; Martásek, P.; Roman, L. J.; Poulos T. L.; Silverman, R. B. *J. Am. Chem. Soc.* **2008**, *130*, 3900-3914.

Ji, H.; Li, H.; Martásek, P.; Roman, L. J.; Poulos, T. L.; Silverman, R. B. *J. Med. Chem.* **2009**, ASAP.

Roman, L. J.; Sheta, E. A.; Martasek, P.; Gross, S. S.; Liu, Q.; Masters, B. S. S. *Proc. Natl. Acad. Sci. U. S. A.* **1995**, *92*, 8428-8432.

Martásek, P.; Liu, Q.; Liu, J. W.; Roman, L. J.; Gross, S. S.; Sessa, W. C.; Masters, B. S. S. *Biochem. Biophys. Res. Commun.* **1996**, *219*, 359-365.

Hevel, J. M.; White, K. A.; Marletta, M. A. *J. Biol. Chem.* **1991**, *266*, 22789-22791.
